# Supplementary material for: Construction of a synthetic methodology-based library and its application in identifying a GIT/PIX protein–protein interaction inhibitor
Source: Nat Commun. 2022 Nov 23;13:7176. doi: 10.1038/s41467-022-34598-7 (PMC9684509; doi:10.1038/s41467-022-34598-7)
Supplement: Supplementary file 1 — Supplementary Info [file 41467_2022_34598_MOESM1_ESM.pdf]

Supplementary Information

1. Supplementary Methods

2. Supplementary Figures 1-8

3. Supplementary Tables 1-4

4. Supplementary References

1. Supplementary Methods

General methods of chemistry

Unless otherwise noted, all reagents and solvents were purchased from commercial sources including Sigma (USA), Aldrich (USA), J&K (China), Aladdin (China) et al. ^{1}H-NMR and ^{13}C-NMR data were obtained on an Agilent 600 MHz operating at 600 MHz and 150 MHz, respectively. Chemical shifts were reported in ppm from tetramethylsilane with the solvent resonance as the internal standard in $CDCl_3$. Data are presented as follows: chemical shift, integration, multiplicity (br = broad, s = singlet, d = doublet, t = triplet, q = quartet, m = multiplet) and coupling constant in Hertz (Hz). MS data were generated on a Waters SQ Detector. HR-MS data were obtained on a Varian 7.0T FTMS.

Representative synthesis work

Synthesis of **15-4-26** (new compound)

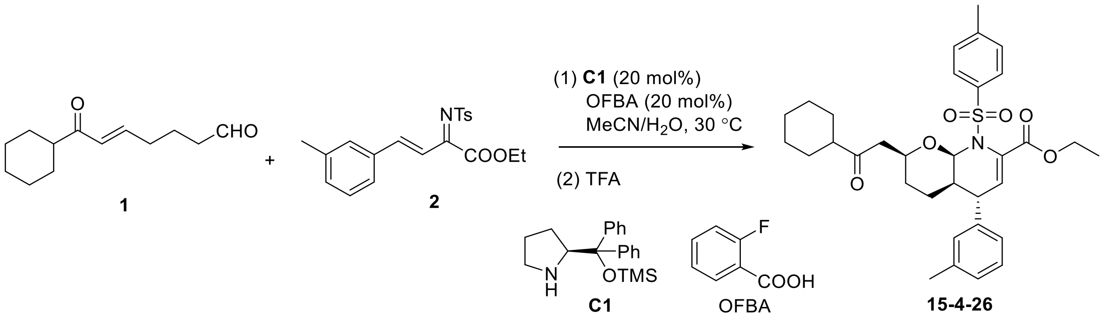

15-4-26 was synthesized according to the procedures reported by our group in 2012 (1). Briefly, the reaction was carried out with aldehyde **1** (2 mmol) and 1-azadiene **2** (1 mmol) in the presence of catalyst **C1** (68 mg, 0.2 mmol) and OFBA (28 mg, 0.2 mmol) in MeCN/H_2O (10/1, 1 mL) at 30 ºC. After completion (monitored by TLC analysis), trifluoroacetic acid (1 mL) was added. The reaction was continued for another 2 h until completion (monitored by TLC analysis). Then the solvents were removed, and the residue was purified by flash chromatography on silica gel (petroleum ether/ethyl acetate) to give fused heterocycle **15-4-26**.

ethyl (2*S*,4a*R*,5*S*,8a*R*)-2-(2-cyclohexyl-2-oxoethyl)-5-(*m*-tolyl)-8-tosyl-3,4,4a,5,8,8a-hexahydro-2*H*-pyrano[2,3-*b*]pyridine-7-carboxylate (15-4-26): 60% yield; 92% ee, determined by HPLC analysis [Daicel chiralcel IA, n-hexane/*i*PrOH = 40/60, 1.0 mL/min, λ = 220 nm, t(major) = 5.52 min, t(minor) = 6.49 min; $[\alpha]_D^{25}$ = +31.2 (0.1 M in $CHCl_3$); mp: 145.0–147.2 °C; ^{1}H-NMR (600 MHz, $CDCl_3$) δ: 7.90 (d, *J* = 7.8 Hz, 2H), 7.33 (d, *J* = 7.8 Hz, 2H), 7.18 (t, *J* = 7.8 Hz, 1H), 7.06 (d, *J* = 7.2 Hz, 1H), 6.91-6.85 (m, 3H), 6.15 (d, *J* = 3.0 Hz, 1H), 4.21 (q, *J* = 7.2 Hz, 2H), 4.09 - 4.04 (m, 1H), 3.47 (dd, *J* = 10.8, 3.2 Hz, 1H), 3.07 (dd, *J* = 10.2, 6.0 Hz, 1H), 2.73 (dd, *J* = 10.2, 6.6 Hz, 1H), 2.44

(s, 3H), 2.33 (s, 3H), 2.25-2.20 (m, 4H), 1.80-1.60 (m, 9H), 1.50-1.25 (m, 4H), 1.21 (t, *J* = 7.2 Hz, 3H); ^{13}C-NMR (150 MHz, CDCl3) δ: 198.3, 164.5, 143.7, 140.9, 140.8, 139.4, 138.4, 136.8, 129.5, 129.1, 128.5, 128.3, 128.0, 127.4, 126.1, 125.8, 84.8, 75.9, 61.4, 43.3, 39.4, 38.7, 36.9, 29.7, 26.1, 25.6, 24.5, 23.0, 21.9, 21.6, 21.5, 21.4, 13.9; IR (neat): νmax 3119, 3056, 2997, 1736, 1704,1653, 1601, 1490, 1459, 1379, 1353, 1233, 1182, 1151, 1038, 946, 871, 823, 753 cm^{-1}; ESI-HRMS calcd for $[C_{33}H_{41}NO_6S + Na]^+$ 602.2547, found 602.2543.

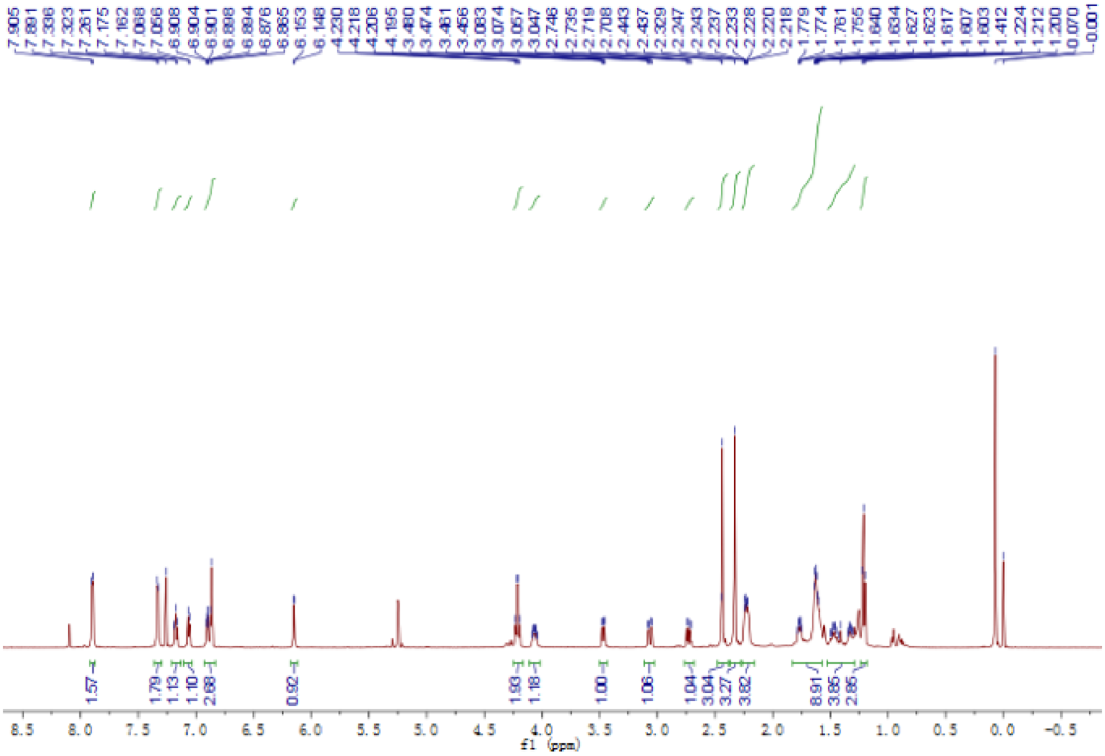

^{1}H-NMR spectrum

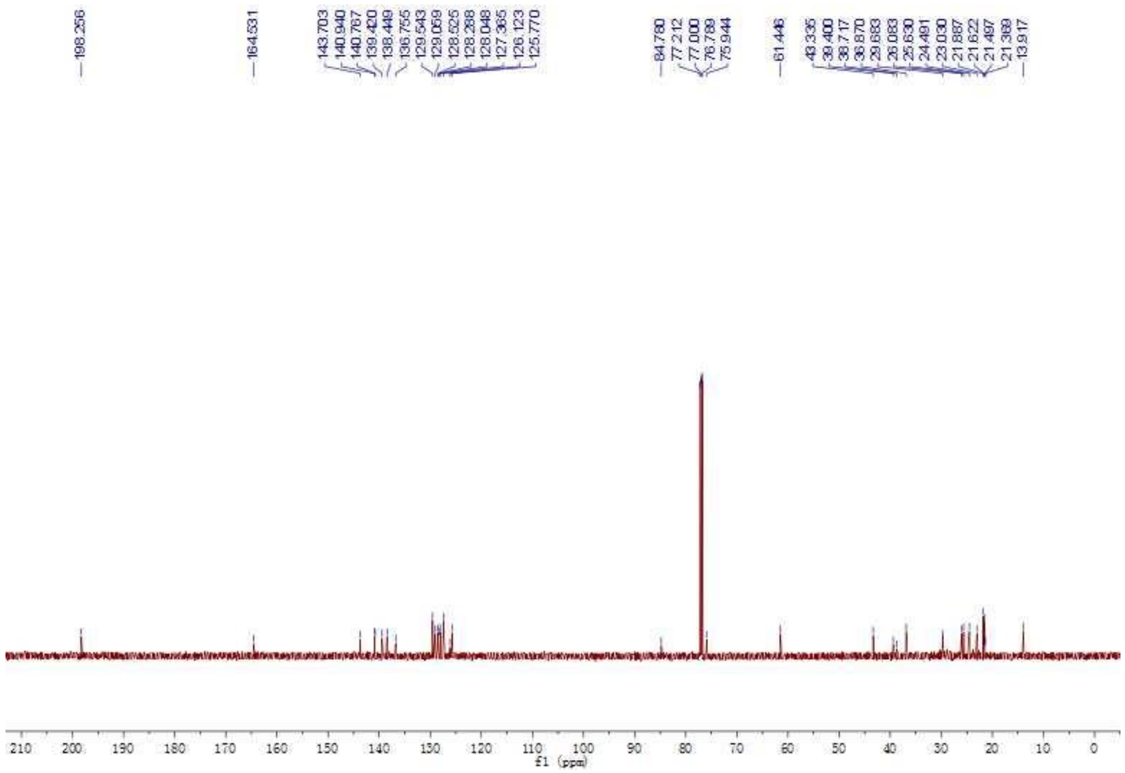

^{1}H-NMR spectrum

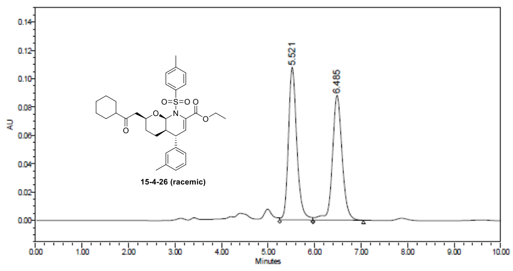

	RT	Area	% Area	Height
1	5.521	1349717	51.29	107969
2	6.485	1282005	48.71	88151

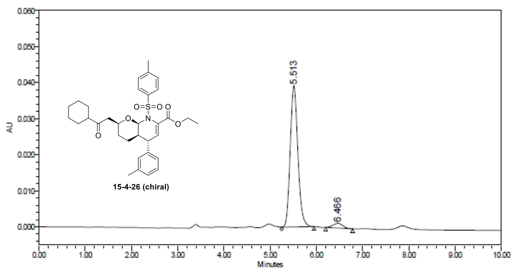

	RT	Area	% Area	Height
1	5.513	450258	95.87	39237
2	6.466	19407	4.13	1290

HPLC spectrum

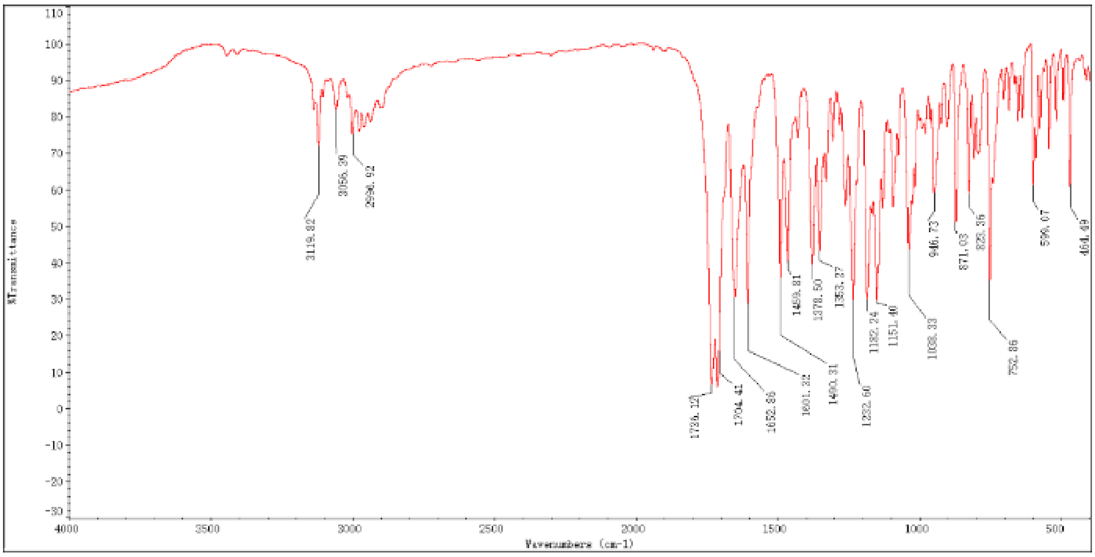

IR spectrum

Synthesis of **14-5-18** (published compound)

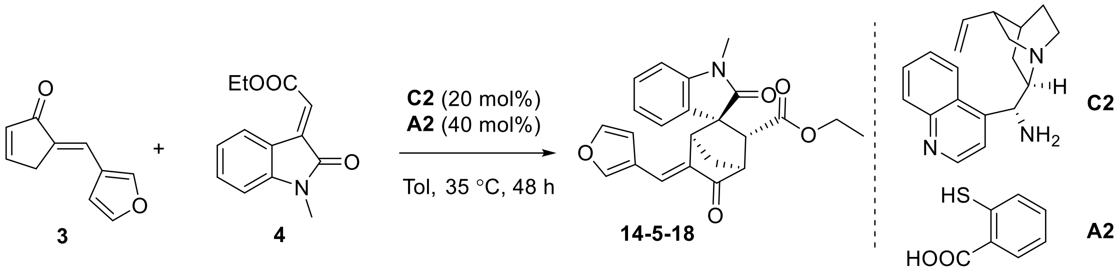

14-5-18 was synthesized following the procedures reported by our group in 2017 (2). The reaction was carried out with **3** (1.0 mmol) and **4** (0.5 mmol), amine **C2** (0.1 mmol) and 2-mercaptobenzoic acid **A2** (0.2 mmol) in dry toluene (5.0 mL) were stirred at 35 ºC until consumption of **4**, which was monitored by TLC analysis. The solvents were removed, and the residue was purified by flash chromatography on silica gel (petroleum ether/ethyl acetate) to give the product **14-5-18**.

ethyl (1*S*,2*R*,3*R*,4*S*,*E*)-6-(furan-3-ylmethylene)-1'-methyl-2',5-dioxospiro [bicyclo[2.2.1]heptane-2,3'-indoline]-3-carboxylate (14-5-18): 83% yield; ^{1}H-NMR (600 MHz, $CDCl_3$): δ (ppm) 7.54 (s, 1H), 7.39-7.30 (m, 3H), 7.07 (t, *J* = 7.2 Hz,1H), 6.94 (d, *J* = 7.2 Hz, 1H), 6.17 (s, 1H), 3.76-3.64 (m, 2H), 3.35 (s, 1H), 3.29 (m, 1H), 3.26 (m, 1H), 3.18 (s, 3H), 2.88 (d, *J* = 10.8 Hz, 1H), 2.04 (d, *J* = 10.8 Hz, 1H), 0.66 (t, *J* = 7.2 Hz, 3H). The ^{1}H-NMR spectrum confirmed that the **14-5-18** was consistent with the compound **4r** in the original paper.

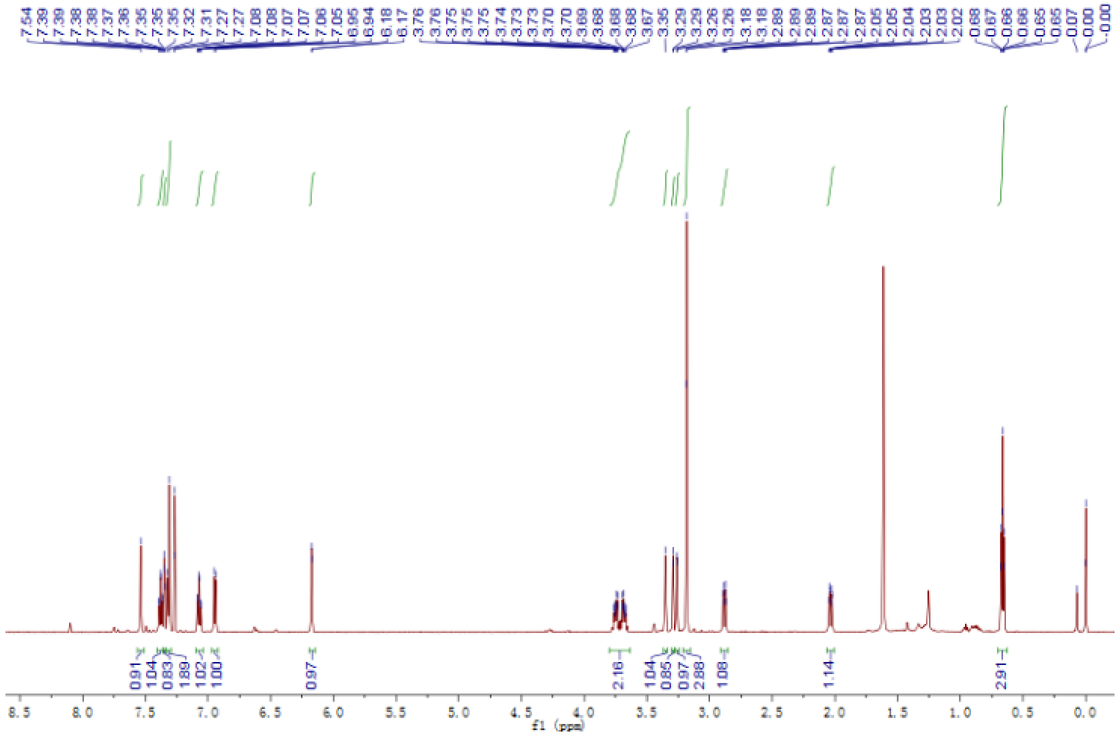

^{1}H-NMR spectrum

2. Supplementary Figures 1-8

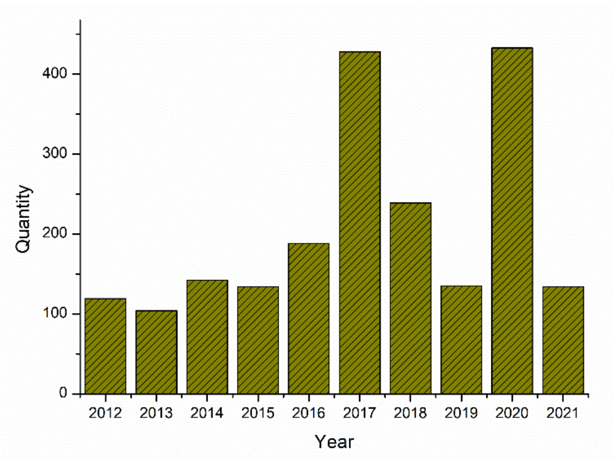

Supplementary Fig. 1 The incoming compound quantity of the entity library each year during 2012-2021. The published articles contributing to construct virtual library (Year 2008-2021) are listed as Supplementary References (1-144).

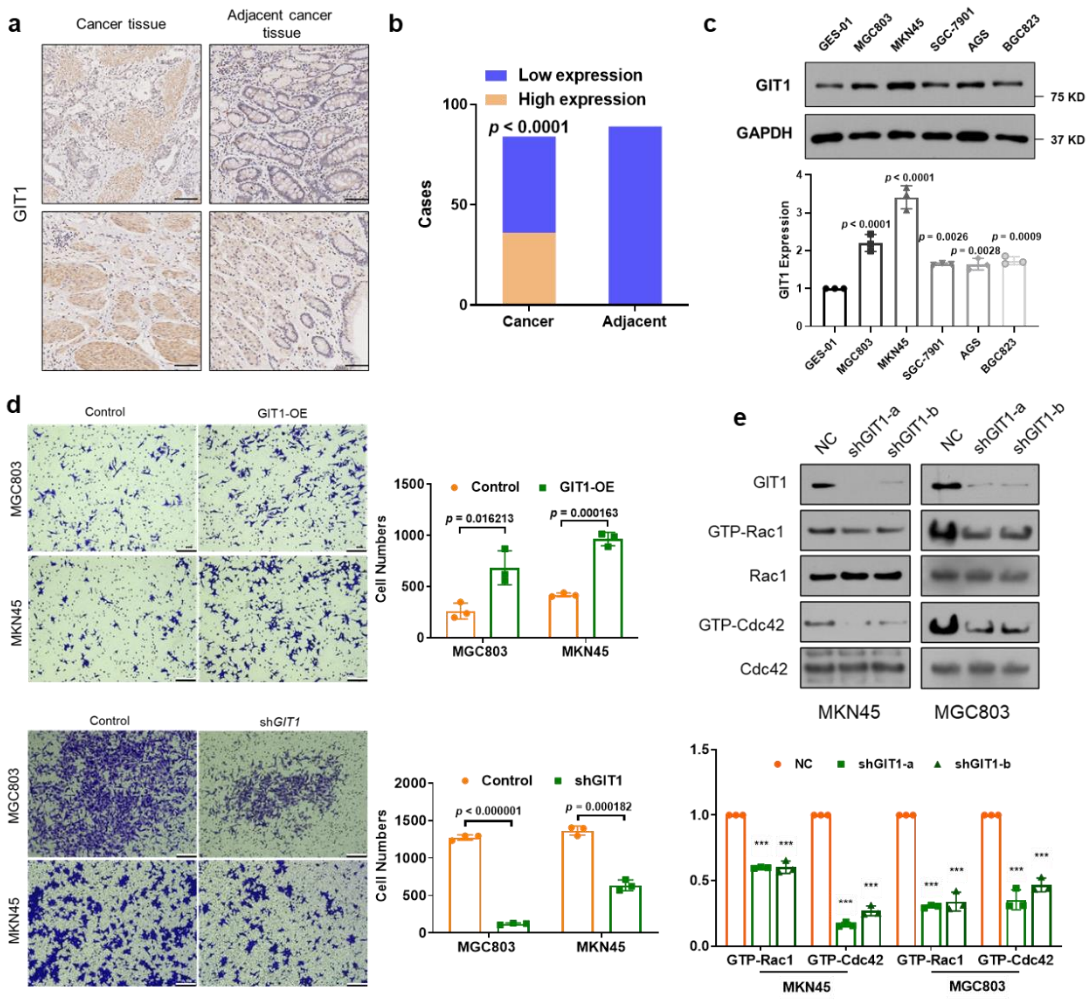

Supplementary Fig. 2 GIT1 is a vital regulating factor in the metastasis of gastric cancer. a & **b** GIT1 protein expression on a tissue microarray constructed from patients' cancer tissues (n = 84) and adjacent cancer tissues (n = 89). The representative images of IHC staining (**a**) and statistic graph of GIT1 expression levels in cancer and adjacent cancer tissues (**b**). Scale bar = 100 µm. **c** GIT1 expression in epithelial cell line (GES-01) and cancer cell lines (MGC803, MKN45, SGC-7901, AGS, BGC823) and the expression level was compared (n = 3 biologically independent samples). **d** Transwell assay to detect the invasion abilities of MGC803 and MKN45 cells after GIT1 overexpression (GIT1-OE) or knock down using a lentiviral short hairpin (sh)RNA (sh*GIT1*). Right columns show the quantification, n = 3 biologically independent samples. Scale bar = 100 µm. **e** The cellular activity of Rac1 and Cdc42 was assessed by quantification of the amount of GTP-loaded Rac1/Cdc42 from cellular lysates using GTP-pull down assay, after GIT1 knockdown using two different shRNA (sh*GIT1*-a/b). The lower column shows the quantification, n = 3 biologically independent samples. Data are presented as mean values ± SD, error bars indicate SD. Statistical analysis: Two-sided Fisher's exact test (**b**), One-way ANOVA, Dunnett's multiple-comparisons test (**c** & **e**, each group was compared with the control group), unpaired two tailed

t tests (**d**), ***$p < 0.0001$ in **e**. Source data are provided as a Source Data file.

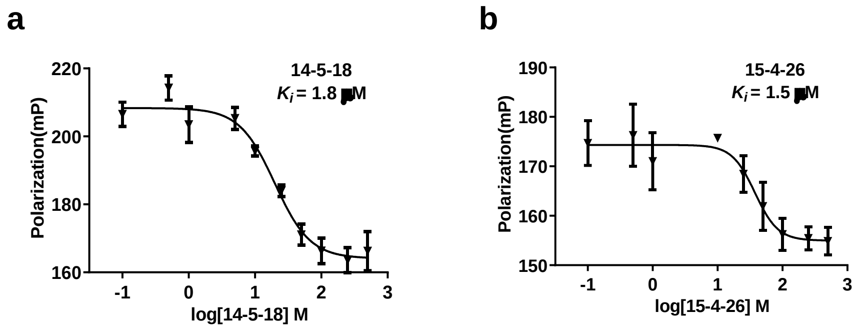

Supplementary Fig. 3 Binding curves of 14-5-18 (a) and 15-4-26 (b) in FP assay. The *Ki* values were calculated according to IC_{50} values of balance. Data are presented as mean values ± SD, error bars indicate SD (n = 3 biologically independent samples). Source data are provided as a Source Data file.

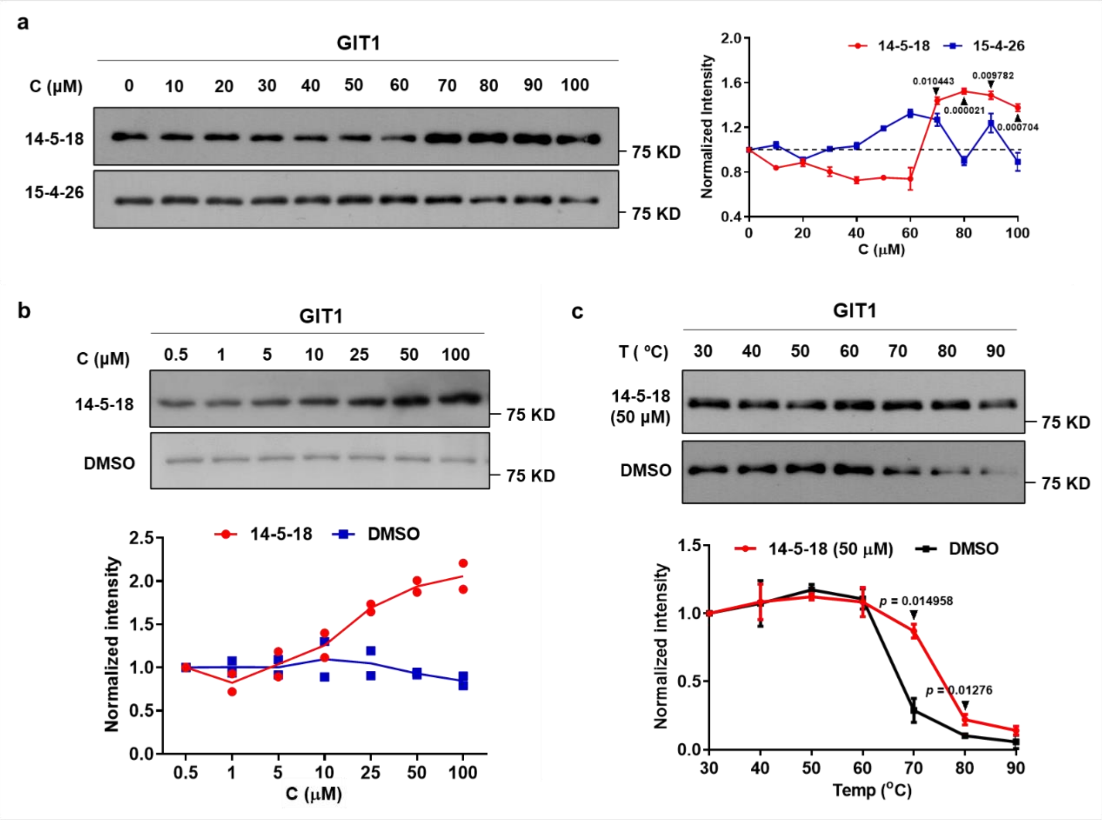

Supplementary Fig. 4 CETSA assays to detect the interaction between 14-5-18 and 15-4-26 with GIT1. Immunoblotting of cell lysates was used to measure the stability of GIT1 protein, when adding the compounds at 0, 10, 20, 30, 40, 50, 60, 70, 80, 90, 100 μM under 65 ºC (**a**), adding **14-5-18** at 0.5, 1.0, 5.0, 10, 25, 50, 100 μM under 65 ºC (**b**), or adding **14-5-18** at 50 μM under 30, 40, 50, 60, 70, 80, 90 ºC (**c**), respectively. The graphs show the quantified results. DMSO was used as solvent control. For **a** and **c**, n = 3 biologically independent samples, for **b**, n = 2 biologically independent samples. Data are presented as mean values ± SD, error bars indicate SD. Statistical analysis: unpaired two tailed *t* tests. Source data are provided as a Source Data file.

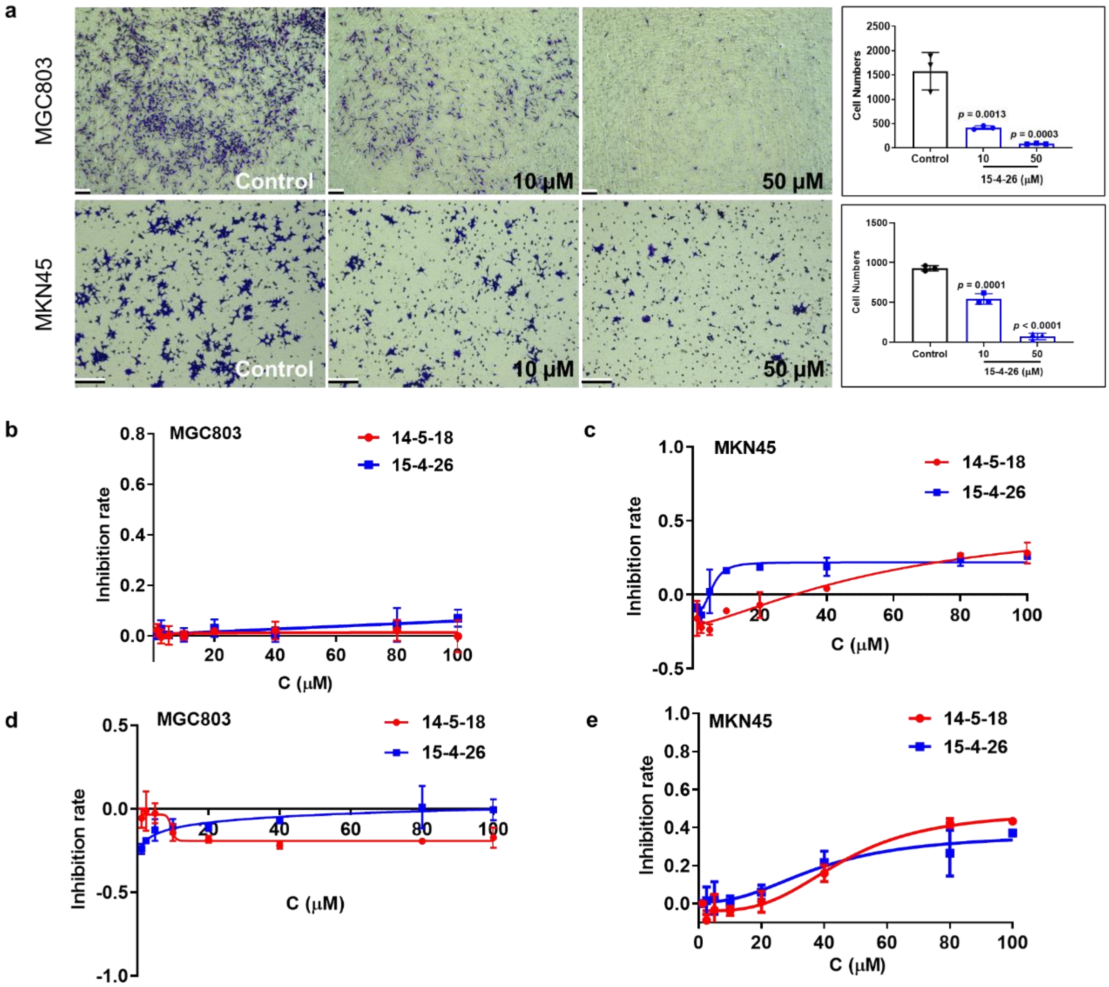

Supplementary Fig. 5 **a** Transwell assay to detect the anti-invasion abilities of **15-4-26** towards MGC803 (upper) and MKN45 (lower) cell lines at 10 and 50 μM for 24 h. The right columns show the respective quantification, n = 3 biologically independent samples. Scale bar = 100 μm. **b** & **c** CCK8 assay was performed to evaluate the anti-proliferation effects of **14-5-18** and **15-4-26** to MGC803 (**b**) and MKN45 (**c**) cell line, at 1.25, 2.5, 5, 10, 20, 40, 80, 100 μM for 24 h (n = 6 biologically independent samples). **d** & **e** CellTiterGlo assay was performed to measure the ATP level of the MGC803 (**d**) and MKN45 (**e**) cell line when treating with **14-5-18** and **15-4-26** at 1.25, 2.5, 5, 10, 20, 40, 80, 100 μM for 24 h (n = 6 biologically independent samples). DMSO was used as solvent control. Data are presented as mean values ± SD, error bars indicate SD. Statistical analysis: One-way ANOVA, Dunnett's multiple-comparisons test, each group was compared with the control group. Source data are provided as a Source Data file.

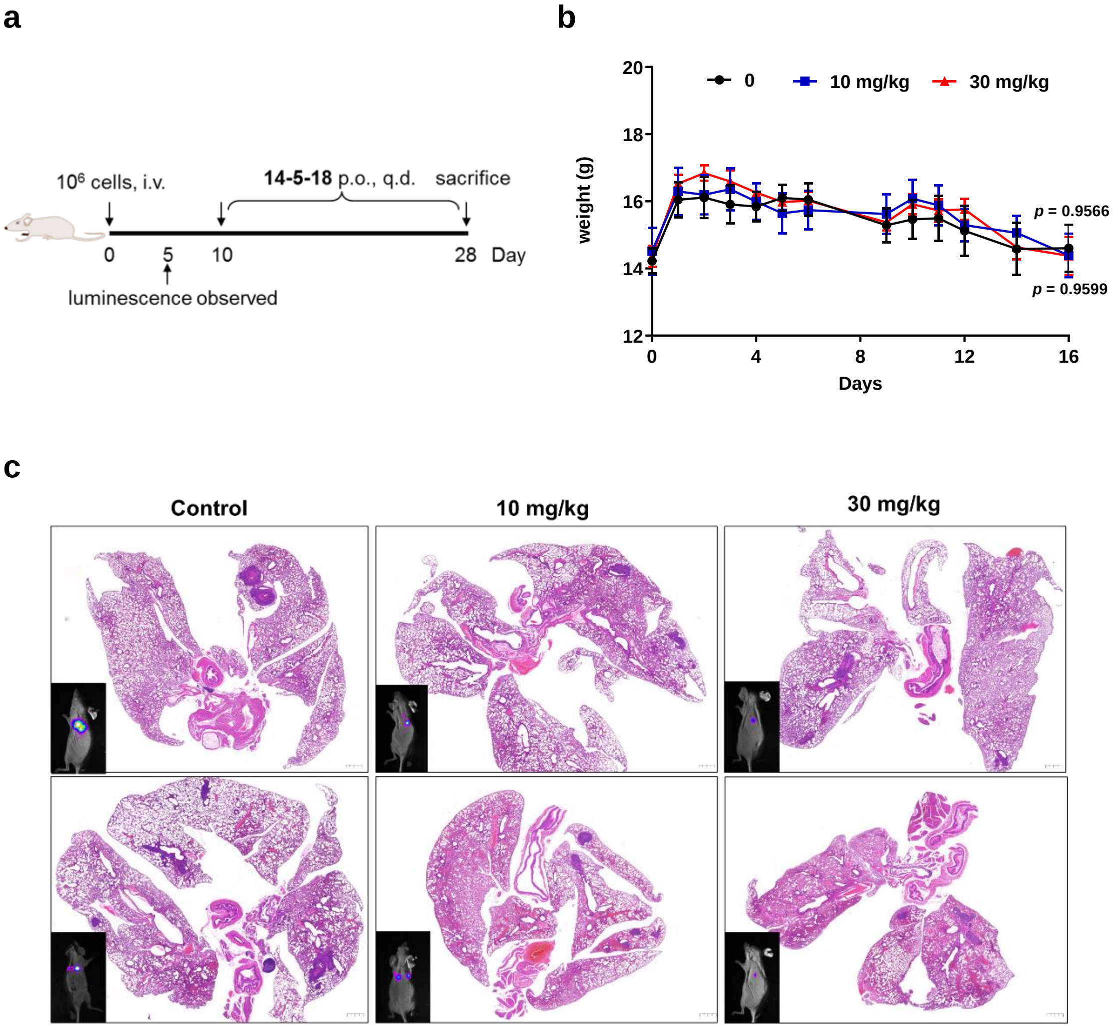

Supplementary Fig. 6 Animal experiments. **a** Scheme of modelling and drug administrating. **b** Curves to show the weight change of mice during experiments (n = 6 biologically independent animals). **c** H & E staining of lung tissues removed from mice at endpoint of the animal experiment, where 2 mice from one group were randomly chosen as representatives to perform the histological staining. Solvent DMSO (2%) + Tween 20 (5%) in water was used as control. Scale bar = 1000 μm. Data are presented as mean values ± SD, error bars indicate SEM. Statistical analysis: One-way ANOVA, Dunnett's multiple-comparisons test, each group was compared with the control group. Source data are provided as a Source Data file.

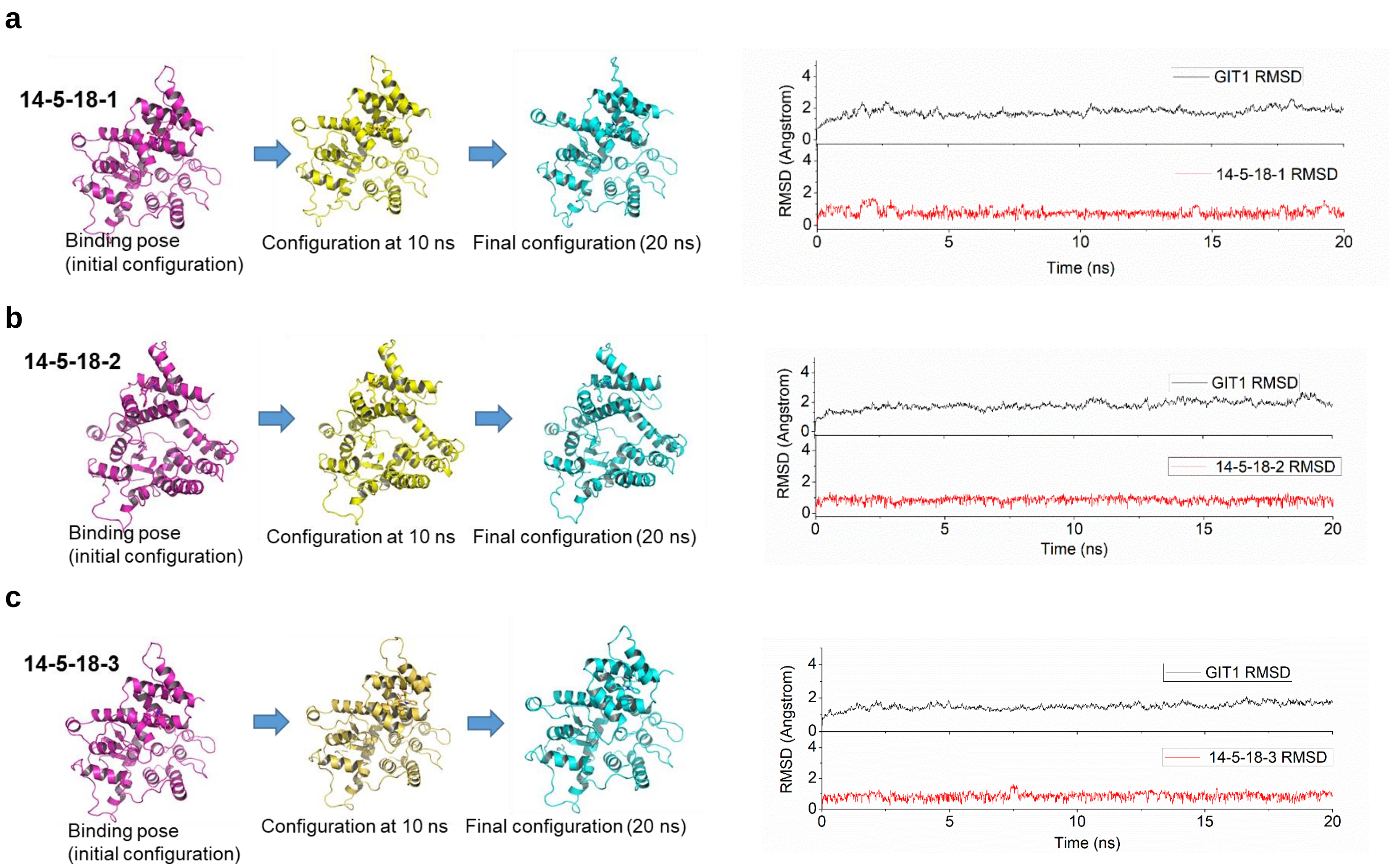

Supplementary Fig. 7 The configuration changes and the root mean square deviation (RMSD) during the molecular dynamic simulation of different binding poses. The configuration changes at time points 0, 10, 20 ns and RMSD of 14-5-18-1 (**a**), 14-5-18-2 (**b**) and 14-5-18-3 (**c**).

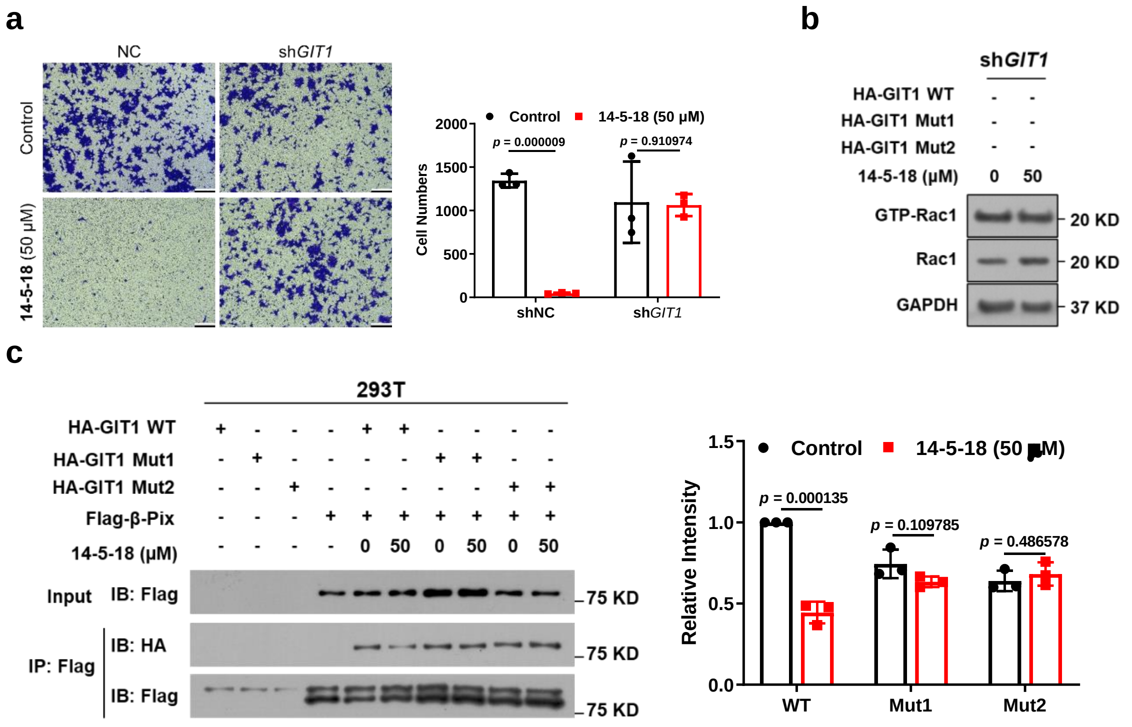

Supplementary Fig. 8 a Transwell assay to detect the anti-invasion ability of **14-5-18** (50 μM) in MKN45 and MKN45-sh*GIT1* cell line. The right column shows the respective quantification, n = 3 biologically independent samples. Scale bar = 100 μm. **b** The cellular activity of Rac1 in MKN45-sh*GIT1* cell line with or without treatment of **14-5-18** (50 μM) for 24 h (The demonstrated figures were representatives from 3 independent experiments with similar results). **c** Co-IP experiments to detect the binding of β-Pix with GIT1 mutants, and the inhibition effects of **14-5-18**. Plasmids expressing HA-GIT1 (wide-type and mutant types $GIT1^{Mut1}$ and $GIT1^{Mut2}$) and Flag-β-Pix were transfected into HEK-293T and the cell lysates were collected to perform the co-IP assay, with or without treatment of **14-5-18** (50 μM). The right column shows the respective quantification, n = 3 biologically independent samples. Otherwise noted, DMSO was used as solvent control. Data are presented as mean values ± SD, error bars indicate SD. Statistical analysis: unpaired two tailed *t* tests. Source data are provided as a Source Data file.

3. Supplementary Tables 1-4

Supplementary Table 1. 2D Fingerprints similarity results for comparison of the commercial libraries and SMBL.

Libraries	Mean Tc (stdev)	% compounds Tc < 0.75	Tc max
SMBL-E vs *chembridge*	0.45 (0.07)	99.93	0.84
SMBL-V vs *chembridge*	0.49 (0.08)	99.61	0.91
targetmol vs *chembridge*	0.66 (0.10)	79.80	1.00
specs vs *chembridge*	0.70 (0.12)	13.49	1.00
SMBL-V vs *targetmol*	0.47 (0.10)	99.29	0.92
SMBL-V vs *specs*	0.50 (0.09)	99.10	0.94
SMBL-E vs *targetmol*	0.52 (0.10)	99.82	0.83
SMBL-E vs *specs*	0.46 (0.08)	99.88	0.85

Supplementary Table 2. *ChemDiv* compounds ranking top 20 in docking and their IC_{50} values in FP assay. --: No FP response at C = 1 mM compound.

Catalog No.	Structure (in smiles)	IC_{50} in FP assay
0443-0206	n1c4c(c(cc1-c2nc3c(cc2)cccc3)C)c5c(cc4)cccc5	--
2578-0155	S(=O)(=O)(NC(C(=O)O)C)c1cc3c(cc1)-c2c(cc(cc2)S(=O)(=O)NC(C(=O)O)C)C3=O	--
3277-0083	c81c(nc(c(n1)NC2C3CC4CC2CC(C3)C4)NC5C6CC7CC5CC(C6)C7)non8	> 100 µM
3277-0261	c71c(n(c(n1)Oc2cc(ccc2)C5C6=C(c3c(ccc4c3cccc4)N5)CC(CC6=O)(C)C)C)C(=O)N(C(=O)N7C)C	> 100 µM
4934-0001	N1(C(=O)C2C(C1=O)CCCC2)C(C(=O)Oc3cc4c(cc3)NC(C=C4C)(C)C)Cc5ccccc5	--
CM4573-7662	S(=O)(=O)(N1C(CC(C1)(F)F)CN3Cc2c(cccc2)CC3)c4c(cccc4)F	--
CM4573-7205	S(=O)(=O)(N1C(CC(C1)(F)F)CN3Cc2c(cccc2)CC3)c4cc(c(cc4)F)Cl	--
D272-0692	C5(=O)N(c1c(ccc(c1)CNC32CC4(CC(C2)(CC(C3)C4)C)C)N5C)C	--
D470-0645	S(=O)(=O)(N2c1nc3c(cc1CC2)ccc(c3)C)c4cc5c(cc4)OCCO5	> 100 µM
D517-1064	c61c([nH]c(c1C2C(=O)N(c3c2cccc3)C)-c4cc5c(cc4)cccc5)NC(=O)NC6=O	--
D638-0006F	c1(nc(nc(c1)Nc2ccc(cc2)NC(=O)Nc3cc(ccc3)SC)C)N(C)C	--
F052-0078	S(=O)(=O)(N1CCCCC1)c4cc2c(nc(cc2)Nc3c(cc(cc3)Br)F)cc4	> 100 µM
G756-2320	N51C(=NC(=CC1=O)COc2cc(ccc2)NC(=O)Nc3cc4c(cc3)OCCO4)C=CC(=C5)Cl	> 100 µM
J030-0445	S(=O)(=O)(c1c(cc3c(c1)C(=O)Nc2c(cccc2)O3)Oc4cc5c(cc4)OCO5)N6CCCC6	--
L111-0329	N2(c1cc(ccc1OCC2=O)S(=O)(=O)CC)Cc3nc(no3)-c4cc5c(cc4)OCO5	--
L676-2486	S(=O)(=O)(c1cc(cc(c1)C)C)N4CCC2(C(=NCCN2)NCc3ccc(cc3)C(F)(F)F)CC4	> 100 µM
L705-0544	S(=O)(=O)(c1cc2c(cc1)SCCC(=O)N2)C(CC(=O)Nc3cc4c(cc3)OCCO4)C	--
S030-1757	N2(C(=O)c1sccc1)CC(C3(C2)CCCCC3)C(=O)NCc4oc(cc4)C	--
S322-0116	c1(cn(c2c1cccc2)C)C7(c3cn(c4c3cccc4)C)CCN(CC5CN(C(=O)C5)c6ccccc6)CC7	--
S646-0838	N1(C(C2(C1)CCCCC2)c3ccncc3)C5CCN(Cc4cnccc4)CC5	--

Supplementary Table 3. The kinetic results for BLI analysis.

Index	Loading Response	Conc. (uM)	Response	KD (M)	KD Error	ka (1/Ms)	ka Error	kdis (1/s)	kdis Error	Full R^2
0	12.30	400	2.1679	7.717E-06	1.026E-07	2.002E02	1.198E00	1.545E-03	1.834E-05	0.9902
1	12.03	200	2.0473	7.717E-06	1.026E-07	2.002E02	1.198E00	1.545E-03	1.834E-05	0.9902
2	12.42	100	1.5608	7.717E-06	1.026E-07	2.002E02	1.198E00	1.545E-03	1.834E-05	0.9902
3	12.43	50	1.0833	7.717E-06	1.026E-07	2.002E02	1.198E00	1.545E-03	1.834E-05	0.9902
4	12.13	25	0.6744	7.717E-06	1.026E-07	2.002E02	1.198E00	1.545E-03	1.834E-05	0.9902

Supplementary Table 4. Components of the binding free energy (kcal/mol) of **14-5-18** docking with GIT1 by different poses calculated by MM/GBSA approach.*

Poses	*E*vdw	*E*ele	*G*gb	*G*np	Δ*G*cal
14-5-18-1	-33.7 ± 2.8	-9.4 ± 4.7	18.7 ± 4.6	-4.1 ± 0.3	-28.6 ± 2.7
14-5-18-2	-37.5 ± 3.8	-24.1 ± 7.0	34.6 ± 6.8	-4.9 ± 0.5	-32.0 ± 3.8
14-5-18-3	-32.5 ± 3.3	-18.3 ± 6.4	28.7 ± 5.5	-4.4 ± 0.3	-26.4 ± 3.7

*The statistical error was estimated based on stabilized 10 ns MD simulation trajectory. 1000 snapshots evenly extracted from the 10-20 ns MD trajectory of complex were used for MM/GBSA calculations and 100 snapshots for the entropy term calculations.

4. Supplementary References

1. Yin, X., Zhou, Q., Dong, L. & Chen, Y. C. Asymmetric sequential aza-Diels-Alder and O-Michael addition: Efficient construction of chiral hydropyrano[2,3-b]pyridines. *Chin. J. Chem*. **30**, 2669 (2012).
2. Zhou, Z. et al. Switchable regioselectivity in amine-catalysed asymmetric cycloadditions. *Nat. Chem*. **9**, 590 (2017).
3. Xiao, B. X. et al. Palladium complex as asymmetric *π*-Lewis base catalyst for activating 1,3-dienes. *J. Am. Chem. Soc.* **143**, 4809 (2021).
4. Liu, B. X., Yan, R. J., Li, X. X., Du, W. & Chen, Y. C. Asymmetric [3+2] annulations of thioaurone and aurone derivatives for the construction of spiroheterocycles. *Asian J. Org. Chem.* **10**, 784 (2021).
5. Lu, J. B. et al. Regioselectivity umpolung in asymmetric Diels–Alder reaction of *ortho*-formyl-substituted cinnamates and dienals via double aminocatalysis. *Org. Lett.* **23**, 145 (2021).
6. Gao, Y., Song, X., Yan, R. J., Du, W. & Chen, Y. C. Asymmetric β, γ′-regioselective [4+3] and [4+2] annulations of α-vinylenals via cascade iminium ion−dienamine catalysis. *Org. Biomol. Chem*. **19**, 151 (2021).
7. Hu, C. X. et al. Construction of enantioenriched 9*H*-fluorene frameworks via cascade reaction involving remote vinylogous dynamic kinetic resolution. *Org. Lett.* **22**, 8973 (2020).
8. Song, X., Yan, R. J., Du, W. & Chen, Y. C. Asymmetric dearomative cascade multiple functionalizations of activated N-alkylpyridinium and N-alkylquinolinium salts. *Org. Lett.* **22**, 7617 (2020).
9. He, Q., Yang, Z. H., Yang, J., Du, W. & Chen, Y. C. Enantioselective formal arylation of (7-aza)isatylidene malononitriles with α′-alkylidene-2-cyclohexenones. *Adv. Synth. Catal.* **362**, 4438 (2020).
10. Liang, S. Y. et al. Phosphine catalyzed enantioselective cascade reaction initiated by intermolecular cross Rauhut–Currier reaction of electron-deficient *ortho*-formyl styrenes. *ChemCatChem* **12**, 5374 (2020).
11. Jiang, Y., Yang, Y., He, Q., Du, W. & Chen, Y. C. Asymmetric intramolecular Rauhut–Currier reaction and its desymmetric version via double thiol/phase-transfer catalysis. *J. Org. Chem.* **85**, 10760 (2020).
12. Xu, C. J., Du, W., Albrecht, Ł. & Chen, Y. C. Lewis basic amine-catalyzed aza-Michael reaction of indole- and pyrrole-3-carbaldehydes. *Synthesis* **52**, 2650 (2020).
13. Ran, G. Y. et al. Cu(I)-catalyzed asymmetric *α*-allenylation of activated ketimines with 3-butynoates. *Org. Lett.* **22**, 4732 (2020).
14. Jiang, B., Du, W. & Chen, Y. C. Modified cinchona alkaloid - catalysed enantioselective [4 + 4] annulations of cyclobutenones and 1-azadienes. *Chem. Commun*. **56**, 7257 (2020).
15. Yan, R. J., Liu, B. X., Xiao, B. X., Du, W. & Chen, Y. C. Asymmetric (4 + 3) and (4 + 1) annulations of isatin-derived Morita−Baylis−Hillman carbonates to construct diverse chiral heterocyclic frameworks. *Org. Lett.* **22**, 4240 (2020).
16. Chen, P., Li, Y., Chen, Z. C., Du, W. & Chen, Y. C. Pseudo-stereodivergent synthesis of enantioenriched tetrasubstituted alkenes via cascade 1,3-oxo-allylation/Cope rearrangement. *Angew. Chem., Int. Ed.* **59**, 7083 (2020).
17. Hu, D., Gao, Y., Song, X., Du, W. & Chen, Y. C. Asymmetric remote addition reactions of heterocycle-based dearomative dienamine or trienamine species to 1-azadienes: Application to construct chiral azocanes and azecanes. *Eur. J. Org. Chem.* **2020***,* 514 (2020).
18. Yang, Y., Jiang, Y., Du, W. & Chen, Y. C. Asymmetric cross [10+2] cycloaddtions of 2-alkylidene-1-indanones and activated alkenes under phase-transfer catalysis. *Chem. Eur. J.* **26**, 1754 (2020).
19. Zheng, P. F. et al. (3 + 1) Annulation/rearrangement cascade of *C,N*-cyclic azomethine imines and 3-chlorooxindoles: Construction of hexahydroindeno[2,1-*c*]pyrazole spirooxindole frameworks. *Org. Lett.* **21**, 10052 (2019).
20. Gao, X. Y. et al. Asymmetric formal vinylogous iminium ion activation for vinyl-substituted heteroaryl and aryl aldehydes. *Org. Lett.* **21**, 9628 (2019).
21. Xiao, B. X. et al. Remote Friedel–Crafts reaction with *α*-heteroaryl-substituted cyclic ketones via HOMO-activation of Lewis bases. *Org. Lett.* **21**, 7554 (2019).

22. Zhou, Z. et al. Double thiol-chiral Brønsted base catalysis: Asymmetric cross Rauhut–Currier reaction and sequential [4 + 2] annulation for assembly of different activated olefins. *Org. Lett.* **21**, 7184 (2019).
23. Chen, Z. C. et al. Cooperative tertiary amine/chiral iridium complex catalyzed asymmetric [4+3] and [3+3] annulation reactions. *Angew. Chem., Int. Ed.* **58**, 15021 (2019).
24. Shi, C. H., Xiao, B. X., Du, W. & Chen, Y. C. Phosphine-catalyzed formal [6 + 2] cycloadditions of α′-methylene 2-cyclopentenones. *Chin. J. Org. Chem.* **39**, 2218 (2019).
25. Chen, L., Xiao, B. X., Du, W. & Chen, Y. C. Quaternary phosphonium salts as active Brønsted acid catalysts for Friedel–Crafts reactions. *Org. Lett.* **21**, 5733 (2019).
26. Ran, G. Y. et al. Asymmetric allylic alkylations with deconjugated carbonyl compounds: Direct vinylogous umpolung strategy. *Angew. Chem., Int. Ed.* **58**, 9210 (2019).
27. Xu, C. J., Li, H. W., He, X. L., Du, W. & Chen, Y. C. Asymmetric direct remote Michael addition reactions of allyl furfurals via dearomative trienamine and tetraenamine catalysis. *Asian J. Org. Chem.* **8**, 1037 (2019).
28. Jiang, B. et al. Sequential assembly of Morita–Baylis–Hillman carbonates and activated *ortho*-vinylbenzaldehydes to construct chiral methanobenzo[7]annulenone frameworks. *Org. Lett.* **21**, 3310 (2019).
29. He, X. L. et al. Asymmetric Barton–Zard reaction to access 3-pyrrole-containing axially chiral skeletons. *ACS Catal.* **9**, 4374 (2019).
30. Zeng, R. et al. [4 + 1 + 1] Annulations of α-bromo carbonyls and 1-azadienes towards fused benzoazaheterocycles. *Org. Lett.* **21**, 2312 (2019).
31. Xiao, B. X., Jiang, B., Song, X., Du, W. & Chen, Y. C. Phosphine-catalysed asymmetric dearomative formal [4 + 2] cycloadditions of 3-benzofuranyl vinyl ketones. *Chem. Commun.* **55**, 3097 (2019).
32. Chen, P. et al. Auto-tandem cooperative catalysis using phosphine/palladium: Reaction of Morita–Baylis–Hillman carbonates and allylic alcohols. *Angew. Chem., Int. Ed.* **58**, 4036 (2019).
33. Yang, Q. Q., Yin, X., He, X. L., Du, W. & Chen, Y. C. Asymmetric formal [5 + 3] cycloadditions with unmodified Morita–Baylis–Hillman alcohols via double activation catalysis. *ACS Catal.* **9**, 1258 (2019).
34. Xiao, B. X., Gao, X. Y., Du, W. & Chen, Y. C. Asymmetric reactions involving Lewis Base catalyst-tethered dearomatizative intermediates. *Chem. Eur. J.* **25**, 1607 (2019).
35. Yan, R. J. et al. Asymmetric dearomative formal [4 + 2] cycloadditions of N,4-dialkylpyridinium salts and enones to construct azaspiro[5.5]undecane frameworks. *Org. Lett.* **20**, 8000 (2018).
36. Chen, Z. C. et al. Organocatalytic enantioselective 1,3-difunctionalizations of Morita–Baylis–Hillman carbonates. *Org. Lett.* **20**, 6279 (2018).
37. Xiao, W., Zhou, Z., Yang, Q. Q., Du, W. & Chen, Y. C. Organocatalytic asymmetric four-component [5+1+1+1] cycloadditions via a quintuple cascade process. *Adv. Synth. Catal.* **360**, 3526 (2018).
38. Duan, C. Q., He, X. L., Du, W. & Chen, Y. C. Asymmetric [4 + 2] cycloadditions with 3-furfural derivatives and α-cyano-α,β-unsaturated ketones. *Org. Chem. Front.* **5**, 2057 (2018).
39. He, X. L. et al. Asymmetric benzylic allylic alkylation reaction of 3-furfural derivatives via dearomatizative dienamine activation. *Chem. Eur. J.* **24**, 6277 (2018).
40. Gu, J. et al. Interrupted Morita–Baylis–Hillman-type reaction of α-substituted activated olefins. *Org. Lett.* **20**, 2088 (2018).
41. He, X. L., Zhao, H. R., Duan, C.-Q., Du, W. & Chen, Y. C. Remote asymmetric oxa-Diels–Alder reaction of 5-allylic furfurals via dearomatizative tetraenamine catalysis. *Org. Lett.* **20**, 804 (2018).
42. Yang, Q. Q., Xiao, W., Du, W., Ouyang, Q. & Chen, Y. C. Asymmetric [4 + 2] annulations to construct norcamphor scaffolds with 2-cyclopentenone via double amine-thiol catalysis. *Chem. Commun.* **54**, 1129 (2018).
43. Xiao, W. et al. Regio- and diastereodivergent [4 + 2] cycloadditions with cyclic 2,4-dienones. *Org. Lett.* **20**, 236 (2018).
44. He, Q., Du, W. & Chen, Y. C. Asymmetric [3+2] annulations to construct 1,2-bispirooxindoles incorporating a dihydropyrrolidine motif. *Adv. Synth. Catal.* **359**, 3782 (2017).
45. Xiao, B. X., Yan, R. J., Gao, X. Y., Du, W. & Chen, Y. C. Asymmetric benzylic

functionalizations of 3-vinyl benzofurans via cascade formal trienamine–vinylogous iminium ion activation. *Org. Lett.* **19**, 4652 (2017).
46. Wang, Z. X. et al. Double activation catalysis for α′-alkylidene cyclic enones with chiral amines and thiols. *Chem. Eur. J.* **23**, 10678 (2017).
47. Li, C., Jiang, K., Liu, T. Y, & Chen, Y. C. Asymmetric [4+1] cycloadditions of *N*-thioacyl Imines and sulfur ylides. *Adv. Synth. Catal.* **359**, 2530 (2017).
48. Zhang, X. R., Zhou, S. L., Yuan, Y., Du, W., & Chen, Y. C. Chemo- and regioselective asymmetric Friedel–Crafts reaction of furans and thiophenes with α,β-unsaturated aldehydes through dual activation. *Synlett* **28**, 1771 (2017).
49. Zhan, G. et al. Direct asymmetric aza-vinylogous-type Michael additions of nitrones from isatins to nitroalkenes. *Chem. Eur. J.* **23**, 6286 (2017).
50. Ran, G. Y. et al. Asymmetric cascade assembly of 1,2-diaza-1,3-dienes and α, β-unsaturated aldehydes via dienamine activation. *Org. Lett.* **19**, 1874 (2017).
51. Xiao, B. X., Du, W. & Chen, Y. C. Asymmetric dearomatizative Diels–Alder reaction for the construction of hydrodibenzo[*b*,*d*]furan frameworks with tetrasubstituted stereogenic centers. *Adv. Synth. Catal.* **359**, 1018 (2017).
52. Lin, W. J., Zhan, G., Shi, M. L., Du, W. & Chen, Y. C. [3+3] formal cycloadditions of nitrones from isatins and azaoxyallyl cations for construction of spirooxindoles. *Chin. J. Chem.* **35**, 857 (2017).
53. Zhou, Z. et al. Cross-conjugated trienamine catalysis with α′-alkylidene 2-cyclohexenones: application in β,γ-regioselective aza-Diels–Alder reaction. *Chem. Eur. J.* **23**, 2945 (2017).
54. Wang, K. K., Du, W., Zhu, J. & Chen, Y. C. Construction of polycyclic spirooxindoles through [3+2] annulations of Morita−Baylis−Hillman carbonates and 3-nitro-7-azaindoles. *Chin. Chem. Lett.* **28**, 512 (2017).
55. Shi, M. L., Zhan, G., Zhou, S. L., Du, W., & Chen, Y. C. Asymmetric inverse-electron-demand oxa-Diels–Alder reaction of allylic ketones through dienamine catalysis. *Org. Lett.* **18**, 6480 (2016).
56. Chen, Y. R., Zhan, G., Du, W. & Chen, Y. C. Regioselective asymmetric formal (3+2) cycloadditions of nitrone ylides from isatins and enals. *Adv. Synth. Catal.* **358**, 3759 (2016).
57. Yang, G. J., Du, W. & Chen, Y. C. Construction of furan derivatives with a trifluoromethyl stereogenic center: Enantioselective Friedel–Crafts alkylations via formal trienamine catalysis. *J. Org. Chem.* **81**, 10056 (2016).
58. Wang, K. K., Wang, P., Ouyang, Q., Du, W. & Chen, Y. C. Substrate-controlled switchable asymmetric annulations to access polyheterocyclic skeletons. *Chem. Commun.* **52**, 11104 (2016).
59. Yuan, X., Zhang, S. J., Du, W. & Chen, Y. C. Asymmetric Diels–Alder cycloadditions of trifluoromethylated dienophiles via trienamine catalysis. *Chem. Eur. J.* **22**, 11048 (2016).
60. Li, C., Jiang, K., Ouyang, Q., Liu, T. Y. & Chen, Y. C. [3 + 1]- and [3 + 2]-Cycloadditions of azaoxyallyl cations and sulfur ylides. *Org. Lett.* **18**, 2738 (2016).
61. Ran, G. Y., Wang, P., Du, W. & Chen, Y. C. α-Regioselective [3 + 2] annulations with Morita–Baylis–Hillman carbonates of isatins and 2-nitro-1,3-enynes. *Org. Chem. Front.* **3**, 861 (2016).
62. He, Q., Zhan, G., Du, W. & Chen, Y. C. Application of 7-azaisatins in enantioselective Morita–Baylis–Hillman reaction. *Beilstein J. Org. Chem.* **12**, 309 (2016).
63. Wang, K. K. et al. *α*-Regioselective asymmetric [3 + 2] annulations of Morita–Baylis–Hillman carbonates with cyclic 1-azadienes and mechanism elucidation. *Org. Lett.* **18**, 872 (2016).
64. Gu, J., Xiao, B. X., Chen, Y. R., Du, W. & Chen, Y. C. Asymmetric Diels–Alder and cascade reaction of quinone imine ketals and 2,4-dienals: Construction of chiral benzo[de]quinolone derivatives. *Adv. Synth. Catal.* **358**, 296 (2016).
65. Zhan, G. et al. Catalyst-controlled switch in chemo- and diastereoselectivities: Annulations of Morita–Baylis–Hillman carbonates from isatins. *Angew. Chem., Int. Ed.* **55**, 2147 (2016).
66. Xiao, W. et al. Asymmetric α, γ-regioselective [3 + 3] formal cycloadditions of α, β-unsaturated aldehydes via cascade dienamine–dienamine catalysis. *Org. Lett.* **18**, 116 (2016).
67. Zhan, G., Shi, M. L., He, Q., Du, W. & Chen, Y. C. [4 + 3] Cycloadditions with bromo-substituted Morita−Baylis−Hillman adducts of isatins and N-(ortho-chloromethyl)aryl

amides. *Org. Lett.* **17**, 4750 (2015).
68. Peng, J., Ran, G. Y., Du, W. & Chen, Y. C. Divergent cyclization reactions of Morita–Baylis–Hillman carbonates of 2-cyclohexenone and isatylidene malononitriles. *Org. Lett.* **17**, 4490 (2015).
69. Li, C., Jiang, K. & Chen, Y. C. Diastereodivergent and enantioselective [4+2] annulations of γ-butenolides with cyclic 1-azadienes. *Molecules* **20**, 13642 (2015).
70. Zheng, P. F. et al. Enantioselective [4+1] annulation reactions of α-substituted ammonium ylides to construct spirocyclic oxindoles. *J. Am. Chem. Soc.* **137**, 9390 (2015).
71. Peng, J., Ran, G. Y., Du, W. & Chen, Y. C. Tertiary amine-catalyzed asymmetric [3 + 2] annulations of Morita–Baylis–Hillman carbonates of isatins with nitroolefins to construct spirooxindoles. *Synthesis* **47**, 2538 (2015).
72. He, X. L., Xiao, Y. C., Du, W. & Chen, Y. C. Enantioselective formal [3 + 3] cycloadditions of ketones and cyclic 1-azadienes via cascade enamine-enamine catalysis. *Chem. Eur. J.* **21**, 3443 (2015).
73. Zhan, G., He, Q., Yuan, X. & Chen, Y. C. Asymmetric direct vinylogous Michael additions of allyl alkyl ketones to maleimides through dienamine catalysis. *Org. Lett.* **16**, 6000 (2014).
74. Zhan, G., Zhou, Q. Q., Du, W. & Chen, Y. C. Enantioselective N-allylic alkylation of propargyl sulfonamides with Morita–Baylis–Hillman carbonates and sequential electrophilic cyclization. *Synthesis* **46**, 3383 (2014).
75. Feng, X., Zhou, Z., Yin, X., Li, R. & Chen, Y. C. Enantioselective direct bisvinylogous 1,6-additions of β-allyl 2-cyclohexenone to α, α-dicyanodienes through trienamine catalysis. *Eur. J. Org. Chem.* **2014**, 5906 (2014).
76. Li, Q. Z., Gu, J. & Chen, Y. C. Organocatalytic asymmetric [4+2] formal cycloadditions of cyclohexenylidenemalononitriles and enals to construct chiral bicyclo[2.2.2]octanes. *RSC Adv.* **4**, 37522 (2014).
77. Gu, J., Ma, C., Li, Q. Z., Du, W. & Chen, Y. C. β, γ-Regioselective inverse-electron-demand aza-Diels–Alder reactions with α, β-unsaturated aldehydes via dienamine catalysis. *Org. Lett.* **16**, 3986 (2014).
78. Zhou, R., Xiao, W., Yin, X. & Chen, Y. C. Diastereo- and enantioselective [4+2] cycloadditions of cyclic enones with cyclic 1-azadienes. *Acta Chim. Sinica*. **72**, 862 (2014).
79. Xiao, Y. C., Yue, C. Z., Chen, P. Q. & Chen, Y. C. Asymmetric dearomatic Diels–Alder reactions of diverse heteroarenes via π-system activation. *Org. Lett.* **16**, 3208 (2014).
80. Yin, X., et al. Asymmetric [5 + 3] formal cycloadditions with cyclic enones through cascade dienamine-dienamine catalysis. *Angew. Chem., Int. Ed.* **53**, 6245 (2014).
81. Li, J. L. et al. Remote enantioselective Friedel–Crafts alkylations of furans via HOMO-activation. *Angew. Chem., Int. Ed.* **53**, 5449 (2014).
82. Zhou, Z., Feng, X., Yin, X. & Chen, Y. C. Direct remote asymmetric bisvinylogous 1,4-additions of cyclic 2,5-dienones to nitroalkenes. *Org. Lett.* **16**, 2370 (2014).
83. Chen, P. Q., Xiao, Y. C., Yue, C. Z. & Chen, Y. C. Trienamine catalysis with linear deconjugated 3,5-dienones. *Org. Chem. Front.* **1**, 490 (2014).
84. Zhou, Q. Q., Xiao, Y. C., Yuan, X. & Chen, Y. C. Asymmetric Diels–Alder reactions of 2,4,6-trienals via tetraenamine catalysis. *Asian J. Org. Chem.* **3**, 545 (2014).
85. Feng, X. et al. Trienamines derived from interrupted cyclic 2,5-dienones: remote δ,ε-C=C bond activation for asymmetric inverse-electron-demand aza-Diels–Alder reaction. *Angew. Chem., Int. Ed.* **52**, 14173 (2013).
86. Ma, C., et al. 1-Azadienes as regio- and chemoselective dienophiles in aminocatalytic asymmetric Diels–Alder reaction. *Org. Lett.* **15**, 6206 (2013).
87. Zhou, Q. Q., Yuan, X., Xiao, Y. C., Dong, L. & Chen, Y. C. Aminocatalytic asymmetric Diels-Alder reaction of phosphorus dienophiles and 2,4-dienals. *Tetrahedron* **69**, 10369 (2013).
88. Liu, J. X., Zhou, Q. Q., Deng, J. G. & Chen, Y. C. An asymmetric normal-electron-demand aza-Diels– Alder reaction via trienamine catalysis. *Org. Biomol. Chem.* **11**, 8175 (2013).
89. Peng, J., Huang, X., Zheng, P. F. & Chen, Y. C. Rauhut–Currier-type reaction with Morita–Baylis–Hillman carbonates of 2-cyclohexenone and alkylidenemalononitriles to access chromene derivatives. *Org. Lett.* **15**, 5534 (2013).
90. Wang, Q. G., Zhou, Q. Q., Deng, J.G. & Chen, Y. C. An asymmetric allylic alkylation-Smiles rearrangement-sulfinate addition sequence to construct chiral cyclic sulfones. *Org. Lett.* **15**, 4786 (2013).
91. Jia, Z. J., Jiang, K., Zhou, Q. Q., Dong, L. & Chen, Y. C. Amine/N-heterocyclic carbene

cascade catalysis for asymmetric synthesis of fused indane derivatives with multiple chiral centres. *Chem. Commun.* **49**, 5892 (2013).
92. Yao, Y., Li, J. L., Zhou, Q. Q., Dong, L. & Chen, Y. C. Enantioselective aza-Morita–Baylis–Hillman reaction with ketimines and acrolein catalyzed by organic assemblies. *Chem. Eur. J.* **19**, 9447 (2013).
93. Zhang, S. J., Zhang, J., Zhou, Q. Q., Dong, L. & Chen, Y. C. Aminocatalytic asymmetric exo-Diels–Alder reaction with methiodide salts of Mannich bases and 2,4-dienals to construct chiral spirocycles. *Org. Lett.* **15**, 968 (2013).
94. Ma, C. et al. A concise assembly of electron-deficient 2,4-dienes and 2,4-dienals: regio- and stereoselective exo-Diels–Alder and redox reaction via sequential amine and carbene catalysis. *Angew. Chem., Int. Ed.* **52**, 948 (2013).
95. Xiao, Y. C., Zhou, Q. Q., Dong, L., Liu, T. Y. & Chen, Y. C. Asymmetric Diels–Alder reaction of 2-methyl-3-indolylmethanols via in situ generation of ortho-quinodimethanes. *Org. Lett.* **14**, 5940 (2012).
96. Feng, X. et al. Stereodivergence in amine-catalyzed regioselective [4+2] cycloadditions of beta-substituted cyclic enones and polyconjugated malononitriles. *J. Am. Chem. Soc.* **134**, 19942 (2012).
97. Sun, X. H. et al. Asymmetric allylic alkylation of cyclic N-sulfonylimines with Morita–Baylis–Hillman carbonates of isatins. *Acta Chimi. Sinica.* 70, 1682 (2012).
98. Zhang, H., Zhang, S. J., Zhou, Q. Q., Dong, L. & Chen, Y. C. Organocatalytic asymmetric allylic amination of Morita–Baylis–Hillman carbonates of isatins. *Beilstein J. Org. Chem.* **8**, 1241 (2012).
99. Li, Q. Z., Ma, L., Dong, L. & Chen, Y. C. Asymmetric aza-Diels–Alder and cation-olefin cyclization sequence: A concise way to fused chiral cyclopenta[b]piperidines. *ChemCatChem* **4**, 1139 (2012).
100. Li, J. L. et al. Asymmetric Diels–Alder reaction of β, β-disubstituted enals and chromone-fused dienes: construction of collections with high molecular complexity and skeletal diversity. *Chem. Sci.* **3**, 1879 (2012).
101. Xiong, X. F. et al. Trienamine catalysis with 2,4-dienones: development and application in asymmetric Diels–Alder reaction. *Angew. Chem., Int. Ed.* **51**, 4401 (2012).
102. Huang, X., Peng, J., Dong, L. & Chen, Y. C. Asymmetric assembly of 2-oxindole and α-angelica lactone units to construct vicinal quaternary chiral centers. *Chem. Commun.* **48**, 2439 (2012).
103. Cui, H. L., Sun, X. H., Jiang, L., Dong, L. & Chen, Y. C. Lewis base-assisted Brønsted base catalysis: direct asymmetric allylic alkylation of indenes. *Eur. J. Org. Chem.* **2011**, 7366 (2011).
104. Zhou, S. L., Li, J. L., Dong, L. & Chen, Y. C. Organocatalytic sequential hetero-Diels−Alder and Friedel−Crafts reaction: constructions of fused heterocycles with scaffold diversity. *Org. Lett.* **2011**, *13*, 5874.
105. Xiao, Y. C., Wang, C., Yao, Y., Sun, J. & Chen, Y. C. Direct asymmetric hydrosilylation of indoles: Combined Lewis base and Brønsted acid activation. *Angew. Chem., Int. Ed.* **50**, 10661 (2011).
106. Peng, J., Huang, X., Jiang, L., Cui, H. L. & Chen, Y. C. Tertiary amine-catalyzed chemoselective and asymmetric [3 + 2] annulation of Morita−Baylis−Hillman carbonates of isatins with propargyl sulfones. *Org. Lett.* **13**, 4584 (2011).
107. Jia, Z. J., Zhou, Q., Zhou, Q. Q., Chen, P. Q. and Chen, Y. C. Exo-selective asymmetric Diels−Alder reaction of 2,4-dienals and nitroalkenes via trienamine catalysis. *Angew. Chem., Int. Ed.* **50**, 8638 (2011).
108. Jiang, L., Lei, Q., Huang, X., Cui, H. L., Zhou, X. & Chen, Y. C. Lewis base assisted Brønsted base catalysis: direct regioselective asymmetric vinylogous alkylation of allylic sulfones. *Chem. Eur. J.* **17**, 9489 (2011).
109. Jia, Z. J. et al. Trienamines in asymmetric organocatalysis: Diels–Alder and cascade reactions. *J. Am. Chem. Soc.* **133**, 5053 (2011).
110. Huang, J. R., Cui, H. L., Lei, J., Sun, X. H. & Chen, Y. C. Organocatalytic chemoselective asymmetric N-allylic alkylation of enamides. *Chem. Commun.* **47**, 4784 (2011).
111. Peng, J., Cui, H. & Chen, Y. C. Organocatalytic asymmetric allylic alkylation of sulfonylimidates with Morita-Baylis-Hillman carbonates. *Sci. Chi. Chem.* **54**, 81 (2011).
112. Xiong, X. F., Zhang, H., Peng, J. & Chen, Y. C. Direct asymmetric Michael addition of cyclic *N*-sulfonylimines to Direct asymmetric Michael addition of cyclic *N*-sulfonylimines to

α, β-unsaturated aldehydes. *Chem. Eur. J.* **17**, 2358 (2011).
113. Han, B., Xiao, Y. C., Yao, Y. & Chen, Y. C. Lewis acid-catalyzed intramolecular direct ene reaction of indoles. *Angew. Chem., Int. Ed.* **49**, 10189 (2010).
114. Peng, J., Huang, X., Cui, H. L. & Chen, Y. C. Organocatalytic and electrophilic approach to oxindoles with C3-quaternary stereocenters. *Org. Lett.* **12**, 4260 (2010).
115. Li, J. L., Kang, T. R., Zhou, S. L., Li, R., Wu, L. & Chen, Y. C. Organocatalytic asymmetric inverse-electron-demand Diels–Alder reaction of electron-deficient dienes and crotonaldehyde. *Angew. Chem., Int. Ed.* **49**, 6418 (2010).
116. He, Z. Q., Zhou, Q., Wu, L. & Chen, Y. C. Asymmetric organocatalytic tandem reaction to chiral pyrimidinone derivatives using urea as dinitrogen source. *Adv. Synth. Catal.* **352**, 1904 (2010).
117. Feng, X., Cui, H. L., Xu, S., Wu, L. & Chen, Y. C. Organocatalytic direct vinylogous Michael addition of α, β-unsaturated γ-butyrolactam to α, β-unsaturated aldehydes and an illustration to scaffold diversity synthesis. *Chem. Eur. J*. **16**, 10309 (2010).
118. Cui, H. L., Jiang, K., Liu, Y. K., Du, W. & Chen, Y. C. Enantioselective direct aldol-type reaction of 4-hydroxycoumarin with ethyl trifluoropyruvate catalyzed by modified cinchona alkaloids. *Chin. Sci. Bull.* **55**, 1732 (2010).
119. Jiang, K., Jia, Z. J., Yin, X., Wu, L. & Chen, Y. C. Asymmetric quadruple aminocatalytic domino reactions to fused carbocycles incorporating a spirooxindole motif. *Org. Lett.* **12**, 2766 (2010).
120. Lei, J., Cui, H. L., Li, R., Wu, L. Ding Z. Y. & Chen, Y. C. Tributyltin hydride-mediated radical cyclisation reactions: efficient construction of multiply substituted cyclopentanes. *Org. Biomol. Chem*. **8**, 2840 (2010).
121. Li, J. L., Zhou, S. L., Han, B., Wu, L. & Chen, Y. C. Aminocatalytic asymmetric inverse-electron-demand aza-Diels–Alder reaction of N-Ts-1-aza-1,3-butadienes based on coumarin cores. *Chem. Commun*. **46**, 2665 (2010).
122. Cui, H. L. et al. Direct asymmetric allylic alkylation of butenolides with Morita-Baylis-Hillman carbonates. *Org. Lett.* **12**, 720 (2010).
123. Jiang, K., Jia, Z. J., Chen, S., Wu, L. & Chen, Y. C. Organocatalytic tandem reaction to construct six-membered spirocyclic oxindoles with multiple chiral centres via a formal [2+2+2] annulation. *Chem. Eur. J.* **16**, 2852 (2010).
124. He, Z. Q., Han, B., Li, R., Wu, L. & Chen, Y. C. Enantioselective construction of lactone[2,3-b]piperidine skeletons via organocatalytic tandem reactions. *Org. Biomol. Chem.* **8**, 755 (2010).
125. Xiong, X. F., Jia, Z. J. Du, W., Jiang, K., Liu, T. Y. & Chen, Y. C. Merging chiral organocatalysts: enantio- and diastereoselective direct vinylogous Mannich reaction of alkylimines. *Chem. Commun.* **2009**, 6994 (2009).
126. Han, B. Xiao, Y. C., He, Z. Q. & Chen, Y. C. Asymmetric Michael addition of γ, γ-disubstituted α, β-unsaturated aldehydes to nitroolefins via dienamine catalysis. *Org. Lett.* **11**, 4660 (2009).
127. Zhang, S. J., Cui, H. L., Jiang, K., Li, R., Ding, Z. Y. & Chen, Y. C. Enantioselective allylic amination of Morita-Baylis-Hillman carbonates catalyzed by modified cinchona alkaloids. *Eur. J. Org. Chem.* **2009**, 5804 (2009).
128. Hu, Z. K., Cui, H. L., Jiang, K. & Chen, Y. C. Enantioselective O-allylic alkylation of Morita-Baylis-Hillman carbonates with oxime. *Sci. China Ser. B: Chem.* **52**, 1309 (2009).
129. Feng, X., Yuan, Y. Q., Cui, H. L., Jiang, K. & Chen, Y. C. Organocatalytic peroxy-asymmetric allylic alkylation. *Org. Biomol. Chem.* **7**, 3660 (2009).
130. Cui, H. L., Feng, X., Peng, J., Lei, J., Jiang, K. & Chen, Y. C. Chemoselective asymmetric N-allylic alkylation of indoles with Morita-Baylis-Hillman carbonates. *Angew. Chem., Int. Ed.* **48**, 5737 (2009).
131. Han, B. et al. Organocatalytic regio- and stereoselective inverse-electron-demand aza-Diels-Alder reaction of α, β-unsaturated aldehydes and N-Tosyl-1-aza-1,3-butadienes. *Angew. Chem., Int. Ed.* **48**, 5474 (2009).
132. Liu, Y. K., Ma, C., Jiang, K., Liu, T. Y. & Chen, Y. C. Asymmetric tandem Michael addition-Wittig reaction to cyclohexenone annulation. *Org. Lett.* **11**, 2848 (2009).
133. Jiang, K., Peng, J., Cui, H. L. & Chen, Y. C. Organocatalytic asymmetric allylic alkylation of oxindoles with Morita-Baylis-Hillman carbonates. *Chem. Commun.* **2009**, 3955 (2009).
134. Li, J. L., Han, B., Jiang, K., Du, W. & Chen, Y. C. Organocatalytic enantioselective hetero-Diels-Alder reaction of aldehydes and o-benzoquinone diimide: Synthesis of optically active hydroquinoxalines. *Bioorg. Med. Chem. Lett.* **19**, 3952 (2009).

135. Cui, H. L. Peng, J., Feng, X., Du, W. Jiang, K. & Chen, Y. C. Dual organocatalysis: Asymmetric allylic-allylic alkylation of α, α-dicyanoalkenes and Morita–Baylis–Hillman carbonates. *Chem. Eur. J*. **15**, 1574 (2009).
136. Han, B., Li, J. L., Ma, C., Zhang, S. J. & Chen, Y. C. Organocatalytic asymmetric inverse electron demand aza Diels–Alder reaction of N-sulfonyl-1-aza-1,3-butadienes and aldehydes. *Angew. Chem., Int. Ed.* **47**, 9971 (2008).
137. Du, W., Liu, Y. K., Yue, L. & Chen, Y. C. Organocatalytic asymmetric 1,3-dipolar cycloaddition of nitrones to nitroolefins. *Synlett* **2008**, 2997 (2008).
138. Liu, Y. K., Liu, H., Du, W., Yue, L. & Chen, Y. C. Reaction control in the organocatalytic asymmetric one pot, three-component reaction of aldehydes, diethyl α-aminomalonate and nitroalkenes: toward diversity-oriented synthesis. *Chem. Eur. J*. **14**, 9873 (2008).
139. Han, B. et al. Discovery of bifunctional thiourea-secondary amine organocatalysts for the highly stereoselective nitro-Mannich reaction of α-substituted nitroacetates. *Chem. Eur. J.* **14**, 8094 (2008).
140. Tian, X. Jiang, K. Peng, J. Du, W. & Chen, Y. C. Organocatalytic stereoselective Mannich reaction of 3-oxindoles. *Org. Lett.* **10**, 3583 (2008).
141. Kang, T. R., Xie, J. W., Du, W., Feng, X. & Chen, Y. C. Stereoselective desymmetrisation of prochiral α, α-dicyanoalkenes via domino Michael-Michael addition reactions. *Org. Biomol. Chem.* **6**, 2673 (2008).
142. Yue, L., Du, W., Liu, Y. K. & Chen, Y. C. Organocatalytic asymmetric direct Michael addition of aromatic ketones to alkylidenemalononitriles. *Tetrahedron Lett.* **49**, 3881 (2008).
143. Zhang, Y., Liu, Y. K., Kang, T. R., Hu, Z. K. & Chen, Y. C. Organocatalytic enantioselective Mannich-type reaction of phosphorus ylides: Synthesis of chiral N-Boc-β-amino-α-methylene carboxylic esters. *J. Am. Chem. Soc.* **130**, 2456 (2008).
144. Chen, W. Du, W., Duan, Y. Z., Wu, Y., Yang, S. Y. & Chen, Y. C. Enantioselective 1,3-dipolar cycloaddition of cyclic enones catalyzed by multifunctional primary amine: beneficial effects of hydrogen bonding. *Angew. Chem., Int. Ed.* **46**, 7667 (2007).
