# Supplementary material for: Construction of a synthetic methodology-based library and its application in identifying a GIT/PIX protein–protein interaction inhibitor
Source: Nat Commun. 2022 Nov 23;13:7176. doi: 10.1038/s41467-022-34598-7 (PMC9684509; doi:10.1038/s41467-022-34598-7)
Supplement: Supplementary file 2 — Reporting Summary [file 41467_2022_34598_MOESM2_ESM.pdf]

nature portfolio

Corresponding author(s): Qin Ouyang
Last updated by author(s): Oct 19, 2022

Reporting Summary

Nature Portfolio wishes to improve the reproducibility of the work that we publish. This form provides structure for consistency and transparency in reporting. For further information on Nature Portfolio policies, see our Editorial Policies and the Editorial Policy Checklist.

Statistics

For all statistical analyses, confirm that the following items are present in the figure legend, table legend, main text, or Methods section.

n/a	Confirmed	
☐	☒	The exact sample size (*n*) for each experimental group/condition, given as a discrete number and unit of measurement
☐	☒	A statement on whether measurements were taken from distinct samples or whether the same sample was measured repeatedly
☐	☒	The statistical test(s) used AND whether they are one- or two-sided<br>*Only common tests should be described solely by name; describe more complex techniques in the Methods section.*
☒	☐	A description of all covariates tested
☐	☒	A description of any assumptions or corrections, such as tests of normality and adjustment for multiple comparisons
☐	☒	A full description of the statistical parameters including central tendency (e.g. means) or other basic estimates (e.g. regression coefficient) AND variation (e.g. standard deviation) or associated estimates of uncertainty (e.g. confidence intervals)
☐	☒	For null hypothesis testing, the test statistic (e.g. *F*, *t*, *r*) with confidence intervals, effect sizes, degrees of freedom and *P* value noted<br>*Give P values as exact values whenever suitable.*
☒	☐	For Bayesian analysis, information on the choice of priors and Markov chain Monte Carlo settings
☒	☐	For hierarchical and complex designs, identification of the appropriate level for tests and full reporting of outcomes
☒	☐	Estimates of effect sizes (e.g. Cohen's *d*, Pearson's *r*), indicating how they were calculated

Our web collection on statistics for biologists contains articles on many of the points above.

Software and code

Policy information about availability of computer code

Data collection	OD values of CCK8 and luminescence intensities of CTG were read using Multi-Mode Detection Platform (SpectraMax Paradigm, Molecular Devices) and analyzed using Microsoft Excel software. Transwell images were collected using fluorescence microscopy (Leica, DMi8) and analyzed using ImageJ software. Immunofluorescence images were obtained using a laser scanning confocal microscope (OLYMPUS FLUOVIEW 1000). Bioluminescence was observed on a Bruker In-Vivo Xtreme system. Biolayer interferometry was performed using ForteBio Octet RED (Sartorius, Germany).
Data analysis	All statistic analyses were performed using GraphPad Prism V8.0 for Windows (acquired from graphpad-prism.cn). Immunofluorescence images were analyzed using ZEN and ImageJ softwares. Immunoblotting bands were analyzed using ImageJ software. Bioluminescence was analyzed using Bruker MI software. OD values of CCK8 and luminescence intensities of CTG were analyzed using Microsoft Excel software.

For manuscripts utilizing custom algorithms or software that are central to the research but not yet described in published literature, software must be made available to editors and reviewers. We strongly encourage code deposition in a community repository (e.g. GitHub). See the Nature Portfolio guidelines for submitting code & software for further information.

Data

Policy information about availability of data

All manuscripts must include a data availability statement. This statement should provide the following information, where applicable:

- Accession codes, unique identifiers, or web links for publicly available datasets
- A description of any restrictions on data availability
- For clinical datasets or third party data, please ensure that the statement adheres to our policy

All data generated in this study are provided in the Source Data file. The protein data of GIT2/β-Pix used in this study is available in PDB database under accession code 6JMT [http://doi.org/10.2210/pdb6JMT/pdb]. The GIT1 structure is available in Alpha Fold under accession code Q9Y2X7 [https://alphafold.com/entry/Q9Y2X7].

Human research participants

Policy information about studies involving human research participants and Sex and Gender in Research.

Reporting on sex and gender	n/a
Population characteristics	n/a
Recruitment	n/a
Ethics oversight	n/a

Note that full information on the approval of the study protocol must also be provided in the manuscript.

Field-specific reporting

Please select the one below that is the best fit for your research. If you are not sure, read the appropriate sections before making your selection.

☒ Life sciences ☐ Behavioural & social sciences ☐ Ecological, evolutionary & environmental sciences

For a reference copy of the document with all sections, see nature.com/documents/nr-reporting-summary-flat.pdf

Life sciences study design

All studies must disclose on these points even when the disclosure is negative.

Sample size	Sample sizes are determined according to previous experience and literatures (see related reference: Jie, M. et al. CircMRPS35 suppresses gastric cancer progression via recruiting KAT7 to govern histone modification. Mol. Cancer 2020, 19, 56).
Data exclusions	No data were excluded.
Replication	For in vitro experiments, the repeats were performed using cells (or cell lysates) from at least 3 wells, each well indicate one repeat. For in vivo experiments, the repeats were performed using 6 mice in each group, one mouse represents one repeat. All attempts at replication were successful.
Randomization	Samples and animals were randomly allocated to groups for in vitro and in vivo experiments.
Blinding	The experiments were not performed in blind. Blinding is not feasible in the experiments for only animals were used in the treatments.

Reporting for specific materials, systems and methods

We require information from authors about some types of materials, experimental systems and methods used in many studies. Here, indicate whether each material, system or method listed is relevant to your study. If you are not sure if a list item applies to your research, read the appropriate section before selecting a response.

Materials & experimental systems

n/a	Involved in the study
	[x] Antibodies
	[x] Eukaryotic cell lines
[x]	Palaeontology and archaeology
	[x] Animals and other organisms
[x]	Clinical data
[x]	Dual use research of concern

Methods

n/a	Involved in the study
[x]	ChIP-seq
[x]	Flow cytometry
[x]	MRI-based neuroimaging

Antibodies

Antibodies used	GIT1, Rabbit polyclonal GIT1, Cell Signaling Technology (#2919), 1:1000 dilution<br>Rac1/2/3, Rabbit polyclonal Rac1 Thr138, Cell Signaling Technology (#2465), 1:1000 dilution<br>Cdc42, Rabbit polyclonal Cdc42 Lys135, Cell Signaling Technology (#2462), 1:1000 dilution<br>GAPDH, Rabbit monoclonal GAPDH (14C10), Cell Signaling Technology (#2118), 1:1000 dilution<br>Myc-Tag, Rabbit monoclonal c-Myc (71D17), Cell Signaling Technology (#2278), 1:1000 dilution<br>HA-Tag, Rabbit monoclonal YPYDVPDYA (C29F4), Cell Signaling Technology (#3724), 1:2000 dilution<br>Flag Tag, Rabbit monoclonal DYKDDDDK (D6W5B), Cell Signaling Technology (#14793), 1:1000 dilution<br>Cy3-labeled Goat Anti-Rabbit IgG (H+L), Beyotime Biotechnology (A0516), 1:800 dilution<br>Goat Anti-Rabbit IgG-HRP, Bio-rad (1706515), 1:3000 dilution<br>Goat Anti-Mouse IgG-HRP, Bio-rad (1706516), 1:3000 dilution
Validation	All antibodies used were validated by the suppliers for their particular application, and also validated in the lab by staining of the cells with or without the specific antigen expression. Validation statements and relevant references for the used antibodies can be found on the manufacturer's website.

Eukaryotic cell lines

Policy information about cell lines and Sex and Gender in Research

Cell line source(s)	Human gastric cancer cell line lines MGC803, MKN45, BGC823, AGS, SGC7901, human gastric epithelial cell line GES-1, and human embryonic kidney cell line HEK-293T were provided by our collaborator, Department of Gastroenterology, Xinqiao Hospital, Third Military Medical University, which were originally purchased from American Type Culture Collection (ATCC), National Collection of Authenticated Cell Cultures (Shanghai, China), or other commercial suppliers.
Authentication	All cell lines were routinely examined and confirmed by the morphology and growing behaviors under microscopy , which are consistent with the respective phenotypes showed by the suppliers. Authentication such as STR profiles of the cell lines could be obtained from the suppliers.
Mycoplasma contamination	All cell lines were tested negative for mycoplasma.
Commonly misidentified lines (See ICLAC register)	SGC7901 is a misidentified cell line registered in ICLAC. As SGC7901 was traditionally considered as a gastric cancer cell line and has been widely used in China, we performed the experiments using SGC7901 as one of the gastric caner cell lines together with other authenticated lines. We believe that the results would not influence the conclusion of the present study. And we can delete the results involving SGC7901 if necessary.

Animals and other research organisms

Policy information about studies involving animals; ARRIVE guidelines recommended for reporting animal research, and Sex and Gender in Research

Laboratory animals	Four-week-old female nude mice were purchased from Vital River Company (Beijing, China) and housed in Specific Pathogen Free (SPF) animal facility (68-71.6°F temperature and 50%-60% humidity) and operated in the Center of Animal Experiments, Third Military Medical University throughout the experiment.
Wild animals	This study did not involve wide animals.
Reporting on sex	Only female nude mice were used in the study.
Field-collected samples	This study did not involve field-collected samples.
Ethics oversight	All animal experiments were approved by the Animal Care Committee of Third Military Medical University. All animal experiments were conducted in compliance with all relevant ethical regulations.

Note that full information on the approval of the study protocol must also be provided in the manuscript.
